# Genome-Wide Analysis of the Hsf Gene Family in *Rosa chinensis* and *RcHsf17* Function in Thermotolerance

**DOI:** 10.3390/ijms26010287

**Published:** 2024-12-31

**Authors:** Yanhui Kang, Pei Sun, Yuan Yang, Maofu Li, Hua Wang, Xiangyi Sun, Wanmei Jin

**Affiliations:** 1 Institute of Forestry and Pomology, Beijing Academy of Agriculture and Forestry Sciences, Beijing 100093, China; kk15110057556@126.com (Y.K.); spfate@126.com (P.S.); yangyuanlgs@126.com (Y.Y.); limaofu2003@163.com (M.L.); happybabyhuahua@163.com (H.W.); 15002407391@163.com (X.S.); 2 Key Laboratory of Biology and Genetic Improvement of Horticultural Crops (North China), Ministry of Agriculture and Rural Affairs, Beijing 100093, China; 3 Beijing Engineering Research Center of Functional Floriculture, Beijing 100093, China; 4 Beijing Engineering Research Center for Deciduous Fruit Trees, Beijing 100093, China

**Keywords:** rose, Hsf family, transcription factor, *RcHsf17*, thermotolerance

## Abstract

Heat shock transcription factors (Hsfs) play an important role in response to high temperatures by binding to the promoter of the heat shock protein gene to promote its expression. As an important ornamental plant, the rose often encounters heat stress during the flowering process. However, there are few studies on the *Hsf* family in roses (*Rosa. chinensis*). In the current study, 19 *Hsf* genes were identified from *R. chinensis* and grouped into three main subfamilies (A, B, and C) according to their structural characteristics and phylogenetic analysis. The expression patterns of *RcHsf* genes were detected in different tissues by quantitative real-time PCR. The *RcHsf* genes exhibited distinct expression patterns at high temperatures, with *RcHsf17* having the highest expression level. *RcHsf17* was localized in the nucleus and had transcriptional activity. The overexpression of *RcHsf17* increased thermotolerance in *Arabidopsis*, suggesting the potential role of *RcHsf17* in the regulation of the high-temperature response. In addition, *RcHsf17* overexpressed in *Arabidopsis* could enhance the response of transgenic *Arabidopsis* to methyl jasmonate. Collectively, this study identified and screened *RcHsfs* in response to high temperatures in roses, providing new insights into the functional divergence of *RcHsfs* and a basis for screening new varieties of rose.

## 1. Introduction

Global warming has led to the frequent occurrence of extremely high temperatures, which has become a major limiting factor affecting the growth and development of horticultural plants [1]. The rose, one of the most famous ornamental plants in the world, has been cultivated for at least 5000 years [2]. High temperatures can cause the petals of roses to curl and even die, which has become a severe challenge for the utilization of rose resources.

Due to the characteristics of anchorage, plants are unable to move to avoid damage when they experience high temperatures, so plants have evolved a systematic regulatory mechanism to cope with high temperatures [1]. Many transcription factors play an important role in plant protection against high temperatures [3,4]. *Hsf* is one of the most important transcription factors in the plant high-temperature response. The expression and activation of *Hsf* genes are strictly regulated by complex regulatory networks [5]. The amount of plant Hsf varies greatly. In *Arabidopsis*, there are 21 *Hsf* genes [6] and 24 *Hsf* genes in tomatoes (*Solanum lycopersicum*) [7]. Thirty-four *Hsf* genes were identified in petunias (*Petunia hybrida*) [8]. Up to 52 *Hsf* genes were identified in Chinese cabbage (*Brassica*) [9]. Although the *Hsf* gene family has considerable differences in size and sequence, it is conserved in structure and function. Typical plant *Hsfs* contain a DNA binding domain (DBD) and an oligomerization domain (OD) at the N terminus. Moreover, a nuclear localization signal (NLS), a nuclear export signal (NES), and a less conserved C-terminal activator domain (CTAD) were also detected in some *Hsfs* [10]. Based on their structure, *Hsfs* can be divided into three major subfamilies (A, B, and C). In general, many subfamily A *Hsfs* act as activators of high temperatures, while subfamily B *Hsfs* act as repressors [11,12]. At high temperature, plants rapidly initiate the transcription and translation of *Hsf* genes, which can bind to the heat shock elements (HSEs) (5′-AGAAnnTTCT-3′) of the hear shock protein (HSP) gene to initiate the expression of a series of genes responding to high temperature [13,14].

The regulatory network of *Hsfs* and HSPs in thermomorphogenesis has been well established in *Arabidopsis* [15]. *HSFA2* plays a key role in regulating the cellular response to various environmental stresses, which has been reported in *Arabidopsis*, tomatoes, and maize [3,16]. *VdHSFA2* and *VvHSFA2* positively regulate the grapevine high-temperature response by regulating their downstream target gene *MBF1c* [12]. MdHSFA8a and the AP2/ERF family member MdAP2.12 interact to activate downstream flavonoid synthesis gene activity and enhance the synthesis accumulation of flavonoid [17]. HSFA6B can interact with bZIP60 and be activated by *bZIP60* to participate in the regulation of maize chlorophyll degradation [18,19]. In barley, the overexpression of *HvHsfA6a* enhances the antioxidant potential of transgenic barley, thereby increasing its heat resistance [20]. LlHsfC2 interacts with LlHsfAs to accelerate their transactivation ability, thereby playing an active role in the high-temperature response in the lily [19]. *Hsf* not only plays an important defense role against abiotic stresses such as high temperatures and drought but also has important functions in plant nitrogen metabolism and leaf, pollen, flower bud, and embryo development [6,21,22]. All these results indicate the diverse and important roles of *Hsfs* in distinct biological processes.

In this study, we identified *R. chinensis Hsf* genes and analyzed the expression patterns of *RcHsf* genes at high temperatures. Based on the expression profile analysis of *RcHsf* genes, *RcHsf17* was found to be a potential effector involved in the high-temperature response. The heat resistance function of *RcHsf17* was examined in *Arabidopsis*. This study provides a basis for studying the function of rose *Hsf* genes in response to various stresses and a new insight for cultivating new heat-resistant varieties of roses.

## 2. Results

### 2.1. Identification and Chromosomal Distribution of Hsf Genes in R. chinensis

**Figure 1:**
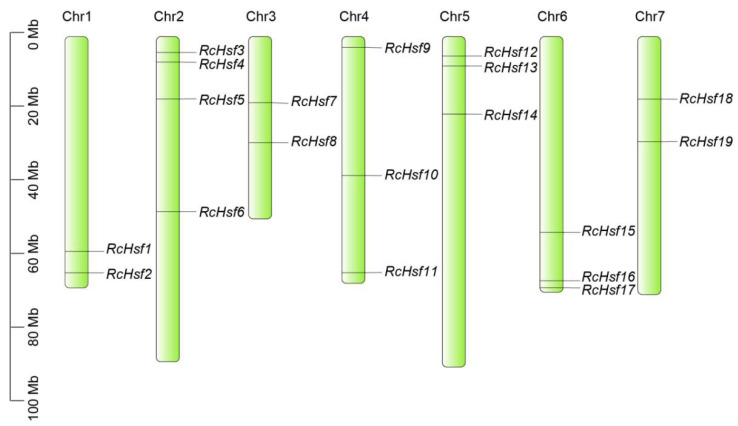
Chromosomal location of *R. chinensis Hsf* family genes. Distribution of 19 *Hsf* genes in *R. chinensis* chromosomes.

The sequences of the putative *RcHsfs* were extracted from the *R. chinensis* genome database using the conserved Hsf domain (PF00447). After filtering and removing the random or segmental duplication, 19 *RcHsfs* were found in the genome database and were almost evenly distributed on chromosome 1 to chromosome 7. *RcHsfs* were distributed on each chromosome, with a maximum of four on chromosome 2. There were three *RcHsfs* on Chr4, Chr5, and Chr6, and two Hsfs were located on Chr1. There was only one *RcHsf* on chromosome 3 and on chromosome 7 (Figure 1). *RcHsf1*, which is not a gene ID found in the *R. chinensis* genome annotation, has a protein annotated as XP_024163952.1 in the NCBI database (Table 1). The physicochemical properties of the 19 *RcHsfs* were analyzed. *RcHsf19* contains 526 amino acids with a maximum molecular weight of 57.12 kD, while *RcHsf12* (129 amino acids) has the smallest molecular weight of 14.71 kD. The isoelectric point (pI) can be used to study the interaction between proteins. When the pIs of two or more protein molecules are close, their interactions may be enhanced. The predicted isoelectric points of the *RcHsfs* ranged from 4.51 (*RcHsf7*) to 9.21 (*RcHsf15*). The details of other parameters are shown in Table 1.

### 2.2. Gene Structure, Phylogeny Analysis, and Conserved Motifs of RcHsf Genes

**Figure 2:**
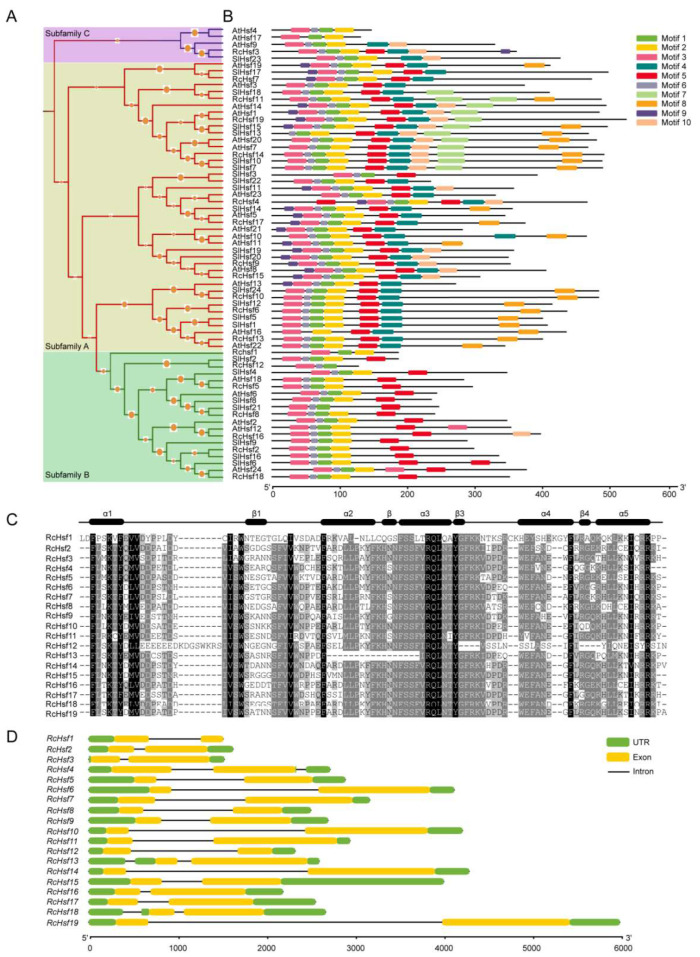
Phylogenetic and structural analysis of RcHsfs. (**A**) The phylogenetic trees of 19 RcHsfs, 24 *Arabidopsis* Hsfs, and 24 tomato (*S. lycopersicum*) Hsfs constructed by the neighbor-joining model in MEGA 5.1. RcHsfs were grouped into three subfamilies: subfamily A (yellow), subfamily B (green), and subfamily C (purple). (**B**) The conserved motif analysis of Hsfs in the rose, *Arabidopsis*, and the tomato (*S. lycopersicum*). Ten motifs are represented by different colors, and their specific sequence information is shown in Figure S1. (**C**) The base sequences and frequencies in the conserved domain of RcHsf proteins. (**D**) Exon and intron structures of 19 *RcHsfs*.

Based on *Arabidopsis* and tomato (*S. lycopersicum*) Hsf family classifications, RcHsfs were grouped into three subfamilies: subfamily A (yellow), subfamily B (green), and subfamily C (purple) (Figure 2A). Consistent with *Arabidopsis* and the tomato (*S. lycopersicum*), the largest number of Hsfs is found in subfamily A in the rose, followed by subfamily B and subfamily C. Subfamily A is the largest with 12 RcHsfs, while there is only one RcHsf in subfamily C (Table 1). Ten conserved motifs, ranging from 6 to 50 amino acids, were identified from RcHsfs using MEME (Figure 2B). Motif 1, motif 2, and motif 3 in the N-terminals represented the characteristic structures of Hsfs (DBD domain) and contained 21, 29, and 29 amino acids, respectively (Figure S1). Specifically, motif 4, referring to the NLS motif, was present in both subfamily A and subfamily C, but not in subfamily B; 13 out of 19 RcHsfs had motif 4. Motif 7 (NES motif) and motif 8 (AHA motif) only existed in subfamily A (Figure 2B). As a typical conserved domain of the Hsf gene, the DBD domain (~80 aa) was widely distributed and highly conserved in most RcHsfs and consisted of five α-helices and four β-sheets (Figure 2C). The structural analysis of the *RcHsfs* showed that most *RcHsfs* contained one intron, while *RcHsf4*, *RcHsf13*, and *RcHsf18* contained two introns (Figure 2D). The above results indicate that the *RcHsfs* are relatively well conserved and may have different functions due to their specific structural characteristics.

### 2.3. Analysis of the Cis-Acting Elements in the RcHsf Promoters

**Figure 3:**
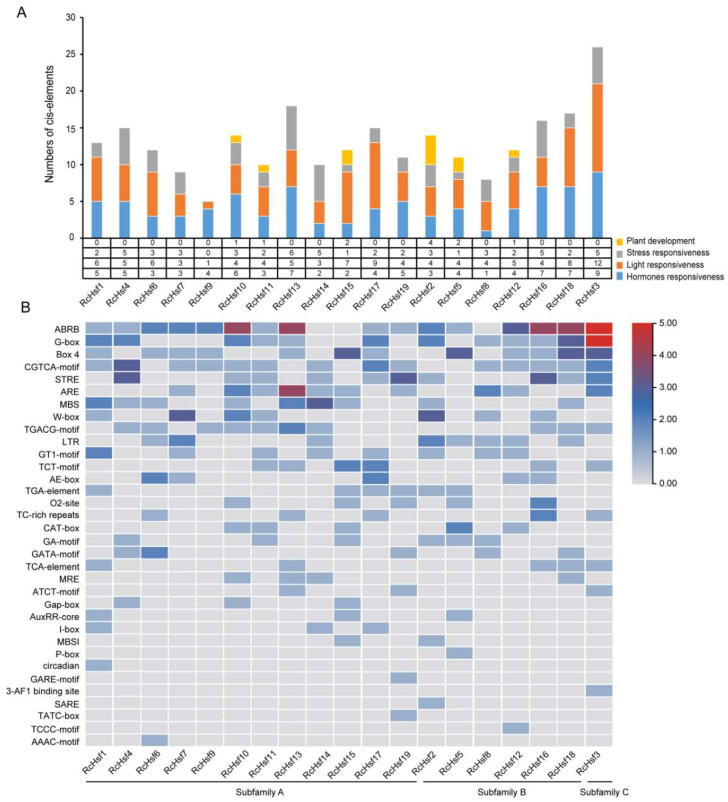
Analysis of the *cis*-acting elements in the promoter sequences of *RcHsf* genes. (**A**) The numbers of *cis*-acting elements in each *RcHsf* gene promoter. The four colors represent four categories: plant development (yellow), stress responsiveness (gray), light responsiveness (orange), and hormone responsiveness (blue). The number of every category was represented by a histogram and listed in the table. (**B**) The heatmap of the number of cis-acting elements in the promoter sequences of the *RcHsf* genes. The color scale from gray to red indicates the number of *cis*-acting elements from 1 to 5. From left to right are subfamily A, subfamily B, and subfamily C.

We analyzed the *cis*-acting elements in the 2 kb promoter sequence of 19 *RcHsf* gene translation start sites. In addition to the core promoter and promoter enhancer, 37 *cis*-acting elements were found, mainly including the *cis*-acting elements involved in plant development, stress responsiveness, and light and hormonal responsiveness (Figure 3A). The numbers of the abscisic acid response elements (ABRE) were the largest, with 37, and they existed in 16 *RcHsf* genes except for *RcHsf8*, *RcHsf14*, and *RcHsf15*. The MeJA-responsive elements (TGACG-motif and CGTCA-motif) were detected in 17 *RcHsf* genes except for *RcHsf2* and *RcHsf15* and were the most widely distributed elements. It is speculated that most *RcHsfs* play an important role in JA response. Furthermore, six salicylic acid-responsive elements (SARE and TCA element), nine auxin-responsive elements (TGA-element and AuxRR-core element), and three gibberellin-responsive elements (GARE-motif, P-box, and TATC-box) were detected. Many defensive and stress-responsive elements, such as TC-rich repeats (defense and stress responsiveness), LTR (low-temperature responsiveness), and STRE (stress responsiveness) were found. In addition, the binding sites of MYB, MYC, and WRKY were observed in the promoter of *RcHsfs* (Figure 3B).

The distribution of *cis*-acting elements was different in subfamily A, subfamily B, and subfamily C (Figure 3A). In subfamily A, the number of *cis*-acting elements was in the range of 5 to 20, and the distribution of *cis*-acting elements was related to plant development, stress responsiveness, hormone responsiveness, and light responsiveness. *RcHsf13* had the most cis-acting elements, and *RcHsf9* had the lowest. In the number and distribution of *cis*-acting elements, subfamily B was similar to subfamily A. *RcHsf16* had the most cis-acting elements, and *RcHsf8* had the lowest. The numbers of *cis*-acting elements were 21 and 9, respectively. In subfamily C, *RcHsf3* had the most *cis*-acting elements, including hormone responsiveness, and the number of light responsiveness elements was larger. The distribution of some elements was specific to the subfamilies. Gap-box, I-box, GARE-motif, TATC-box, and AAAC-motif were only distributed in the promoter of subfamily A, while P-box and TCCC-box were only distributed in subfamily B (Figure 3B). The difference in the *cis*-acting elements in terms of *RcHsf* promoters indicated that *RcHsfs* may play different roles in plant development and stress responses.

### 2.4. Expression of RcHsf Genes in Different Tissues

**Figure 4:**
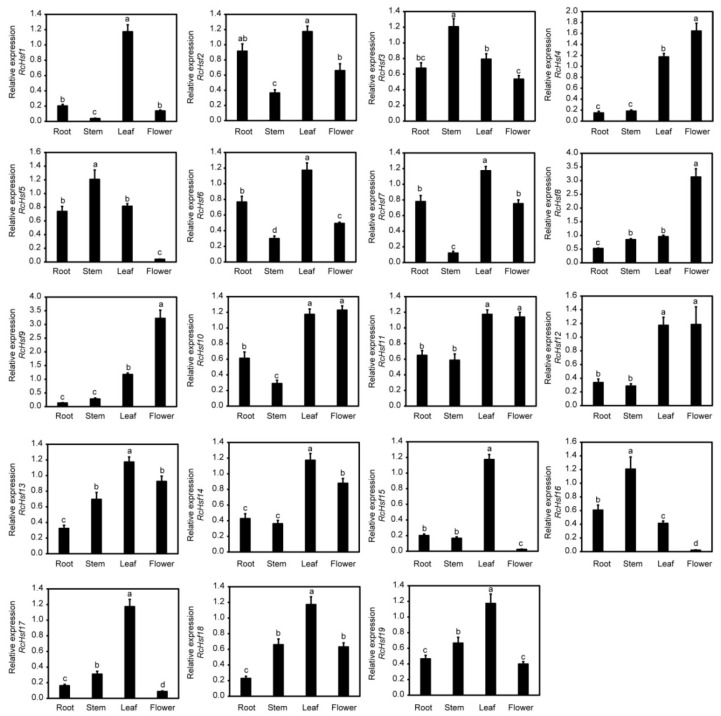
The expression of *RcHsf* genes in rose roots, stems, leaves, and flowers. The letters above the bar charts indicate the significant differences between different samples (*p* < 0.05).

To explore *RcHsf* gene expression specificity, we analyzed the expression profile of *RcHsf* genes in rose roots, stems, leaves, and flowers. As shown in Figure 4, among the 19 *RcHsf* genes, 14 *RcHsf* genes were expressed at relatively high levels in the leaves. *RcHsf3*, *RcHsf5*, and *RcHsf16* were highly expressed in the stems, while *RcHsf1*, *RcHsf2*, *RcHsf4*, *RcHsf6*, *RcHsf7*, *RcHsf12*, and *RcHsf14* were lowly expressed in the stems. Among them, *RcHsf16* was lowly expressed and almost undetectable in the flowers. The expression of *RcHsf4*, *RcHsf8*, and *RcHsf9* in the flowers was significantly higher than that in the roots, stems, and leaves. In general, most of the *RcHsf* genes were expressed in the roots, stems, leaves, or flowers. There was no significant tissue-specific difference in the expression of the three subfamily *RcHsf* genes in the different tissues.

### 2.5. Expression Analyses of RcHsf Genes at High Temperatures

**Figure 5:**
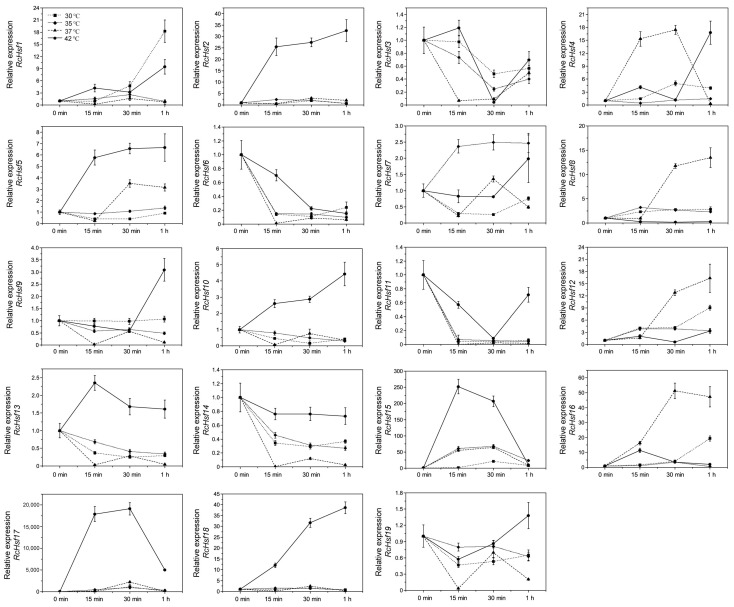
The expression of *RcHsf* genes at high temperatures. The expression of *RcHsf* genes at 30 °C, 35 °C, 37 °C, and 42 °C for 15 min, 30 min, and 1 h; 25 °C is the control temperature.

In order to investigate the response of *RcHsf genes* to high temperatures, we measured the expression of *RcHsf* genes in rose petals at high temperatures. The results showed that the expression of *RcHsf genes* was significantly different at high temperatures, and not all *RcHsf* genes can be induced by high temperatures. When the treatment temperature was lower than 37 °C, nine *RcHsf* genes (*RcHsf1*, *RcHsf2*, *RcHsf5*, *RcHsf7*, *RcHsf12*, *RcHsf15*, *RcHsf16*, *RcHsf17*, and *RcHsf18*) were induced, and the expression level increased with the increase in temperature (Figure 5). *RcHsf1* expression level significantly increased at 30 °C for 1h, while the highest level of *RcHsf4* and *RcHsf7* expression was observed at 35 °C (Figure 5). *RcHsf12* and *RcHsf16* expression increased at 37 °C compared with other treatments. The expression of *RcHsf3*, *RcHsf6*, *RcHsf11*, and *RcHsf13* decreased at 37 °C, while the expression of *RcHsf9* and *RcHsf14* decreased at high temperatures above 30 °C. *RcHsf17* was rapidly induced at high temperatures above 30 °C (Figure 5). These results indicate that *RcHsf genes* have different response modes to different temperatures, which may be due to their different functions in the high-temperature response process.

Among all *RcHsfs*, *RcHsf17* had the highest expression under the high-temperature treatment. In addition, the expression of *RcHsf17* changed more significantly with the increase in temperature. Therefore, we speculated that *RcHsf17* may be a potential regulatory factor in response to high temperatures in roses.

### 2.6. Spatio-Temporal Expression Patterns of RcHsfA6 and Analysis of Localization and Transcriptional Activity

**Figure 6:**
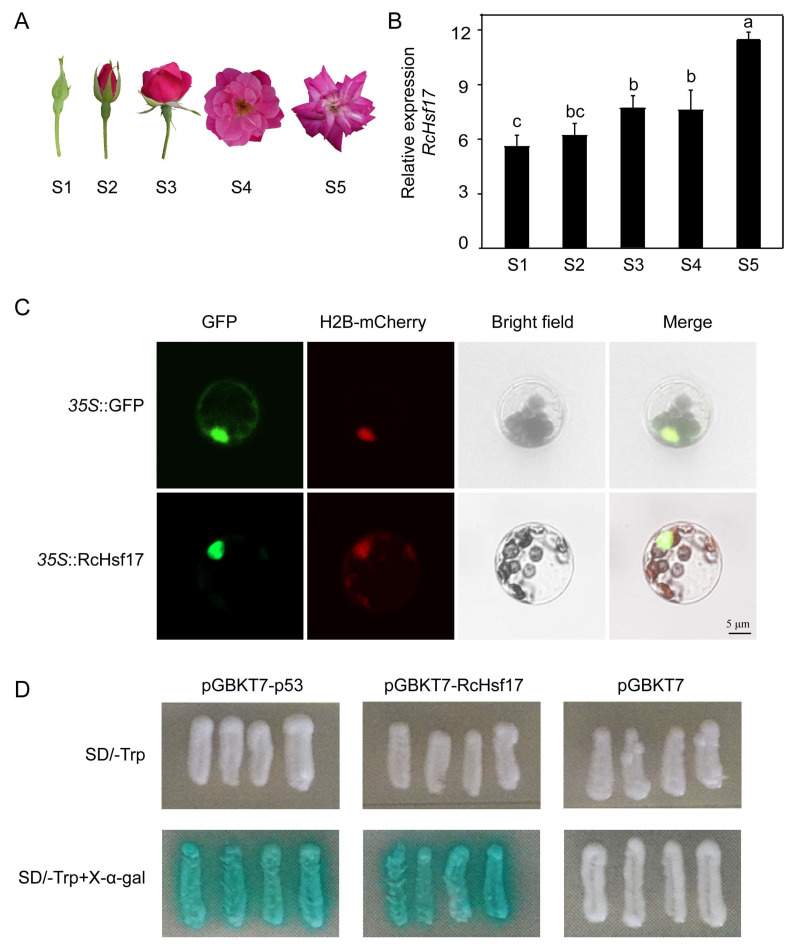
Expression profile analysis and subcellular localization and transcriptional activity assay of *RcHsf17.* (**A**) Phenotypes of the five stages of the flower development process in *R. chinensis*; S1: small bud stage, bud closed; S2: big bud stage, petals slightly exposed; S3: initial opening stage, petals extended; S4: full bloom stage, petals fully open; S5: late blooming stage, petals become curly, and the color becomes lighter. (**B**) Expression of *RcHsf17* in the five stages of the flower development process. (**C**) Subcellular localization of the RcHsf17 protein. GFP: *35S::*RcHsf17 fusion protein; H_2_B-mCherry: H_2_B-mCherry fusion protein (positive marker); Merged: merged image of *35S::*RcHsf17, H_2_B-mCherry, and bright-field. Scale bar: 5 µm. (**D**) Yeast one-hybrid assay. X-α-gal: 5-Bromo-4-chloro-3-indoxyl-α-D-galactopyranoside (the chromogenic substrate of yeast galactosidase); pGBKT7-p53: positive control; pGBKT7: negative control. At least four yeast cells were tested for each construct. The letters above the bar chart indicate the significant difference between the different samples (*p* < 0.05).

To further explore the function of *RcHsf17*, we further analyzed the expression profile of *RcHsf17.* The results showed that *RcHsf17* expression gradually increased with the flower development process, and the expression level was the highest in the late blooming stage (S5) of flower development (Figure 6A,B). The expression profile analysis of *RcHsf17* in different rose tissues showed that *RcHsf17* was highly expressed in leaves, followed by stems, with the lowest expression in roots and petals (Figure 4).

To clarify the localization of RcHsf17, the recombinant plasmids super1300-RcHsf17-GFP (*35S::*RcHsf17) and super1300-GFP (*35S::*GFP) were transformed into *Arabidopsis* protoplasts. The RcHsf17 fusion protein only emitted a green fluorescence signal in the nucleus of the *Arabidopsis* protoplasts, indicating that RcHsf17 played a role in the nucleus (Figure 6C).

The transcriptional activity of RcHsf17 was analyzed by yeast one-hybrid assay. The recombinant plasmid pGBKT7-RcHsf17 was transformed into Y1H gold yeast cells. After 48 h, four monoclonals were streaked and cultured on an SD/-Trp medium containing X-α-gal and SD/-Trp mediums. After 48~72 h, the yeast cells were transformed by pGBKT7-RcHsf17; the positive control became blue, and the negative control did not change (Figure 6D). These results indicate that *RcHsf17* has transcriptional activity.

### 2.7. RcHsf17 Improved Thermotolerance in Transgenic Arabidopsis

**Figure 7:**
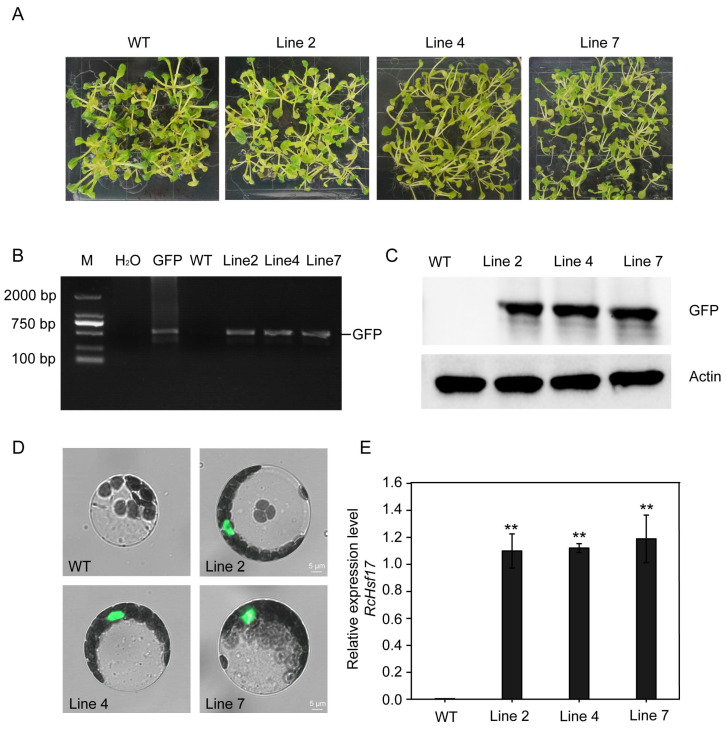
Generation and characterization of *RcHsf17* overexpression transgenic lines. (**A**) *RcHsf17* overexpression lines obtained on a 1/2 MS medium containing 50 mg/L of hygromycin. WT: wild type; Line 2/4/7: *RcHsf17* overexpression transgenic homozygous lines. (**B**) PCR amplification of the GFP gene DNA fragment. M: 2000 DNA marker; GFP: The recombinant plasmid super1300-RcHsf17-GFP; WT: wild type. (**C**) Expression of RcHsf17 protein assayed by Western blot. (**D**) Subcellular localization of RcHsf17 in the protoplasts of the *RcHsf17* overexpression transgenic homozygous line. (**E**) The expression of *RcHsf17* was detected by RT-PCR. ** *p* < 0.01 by *t*-test.

To investigate the function of *RcHsf17*, we heterologously transformed *RcHsf17* into *Arabidopsis*. Three *RcHsf17* overexpression transgenic homozygous lines (Line 2, Line 4, and Line 7) were obtained with 50 mg/L of hygromycin (Figure 7A) and were further identified by PCR and Western blotting (Figure 7B,C). Moreover, we extracted protoplasts of the *RcHsf17* transgenic lines and observed the expression of GFP protein fluorescence. Consistent with the subcellular localization of the RcHsf17 protein, GFP protein fluorescence was observed in the nucleus (Figure 7D). The expression of *RcHsf17* in the transgenic lines was significantly higher than in the wild type (Figure 7E). These results indicate that we have obtained three transgenic homozygous lines with a high expression of *RcHsf17*.

The phenotype of three *RcHsf17* overexpression transgenic lines and the wild type were recorded (Figure S3A). After 10 days of culture on an agar medium, both the transgenic lines and the wild type were able to germinate normally (Figure S3A). Moreover, there was no significant difference in the length of root hairs between the wild type and the transgenic plants (Figure S3B). In addition, there was no difference in the number of rosette leaves between the transgenic lines and the wild type (Figure S3C). These results indicate that the overexpression of *RcHsf17* does not affect the normal growth of *Arabidopsis*.

**Figure 8:**
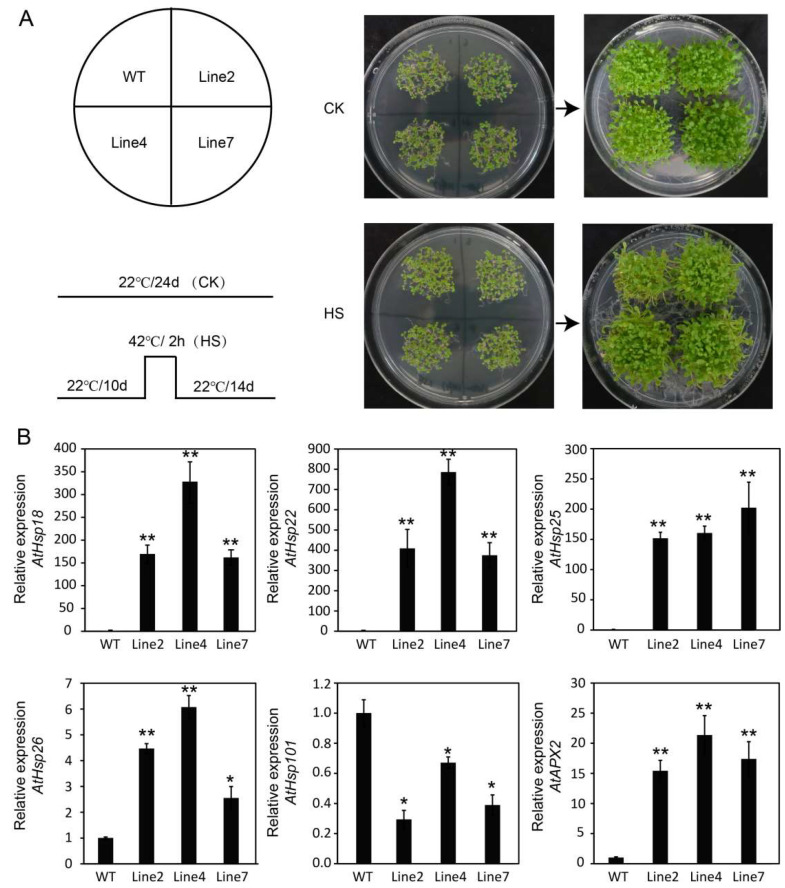
Thermotolerance analyses of *RcHsf17* overexpression in *Arabidopsis* seedlings. (**A**) The phenotype of transgenic and wild-type seedlings. CK: seedlings grown at 22 °C for 24 days; HS: 10-day-old transgenic and wild-type seedlings were treated at 42 °C for 2 h and then cultured at 22 C for 14 d. (**B**) Gene expression related to the high-temperature response, *AtHsp18*, *AtHsp22*, *AtHsp25*, *AtHsp26*, *AtHsp101*, and *AtAPX2* in *RcHsf17* overexpression transgenic lines and wild-type seedlings. Data are means ± SD of three biological replicates. The significant differences between the wild-type and the *RcHsf17* overexpression transgenic lines are shown with asterisks. * *p* < 0.05, ** *p* < 0.01.

**Figure 9:**
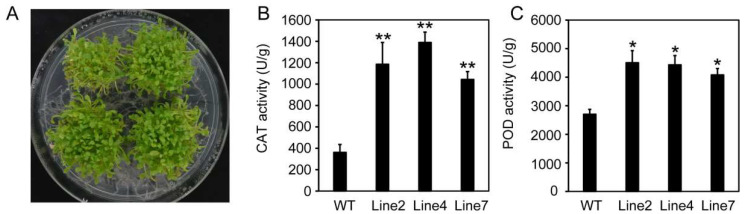
Determination of enzyme activities in wild-type and *RcHsf17* overexpression *Arabidopsis* seedlings. (**A**) The phenotype of transgenic and wild-type seedlings at 42 °C for 2 h. Ten-day-old transgenic and wild-type seedlings were treated at 42 °C for 2 h and then cultured at 22 °C for 14 d. (**B**) CAT activity in wild-type and *RcHsf17* overexpression lines after 42 °C treatment for 2 h. (**C**) POD activity in wild type and *RcHsf17* overexpression lines after 42 °C treatment for 2 h. * *p* < 0.05, ** *p* < 0.01.

In order to verify the role of *RcHsf17* in the high-temperature response, 10-day-old seedlings of wild-type and *RcHsf17* overexpression lines were transferred to an artificial climate chamber adjusted to 42 °C for 2 h (HS), and then the temperature was adjusted to 22 °C to continue growing (Figure 8A). The gene expression related to the high-temperature response in the wild-type and transgenic lines were detected by RT-PCR, including *AtHsp18*, *AtHsp22*, *AtHsp25*, *AtHsp26*, *AtHsp101*, and *AtAPX2*. As shown in Figure 8B, the expression of *AtHsp18*, *AtHsp22*, *AtHsp25*, *AtHsp26*, and *AtAPX2* in the *RcHsf17* overexpression lines was significantly higher than that in the wild type, while there was no significant difference in the expression of *AtHsp101* between the *RcHsf17* overexpression lines and the wild type. Moreover, we detected the expression of *AtHsp* genes in wild-type and transgenic seedlings grown on soil for 15 days by RT-PCR at 42 °C. We found the expression of *AtHsp18*, *AtHsp22*, *AtHsp25*, *AtHsp26*, *AtHsp101*, and *AtAPX2* in *RcHsf17* overexpression lines was significantly higher than that in the wild type at 42 °C (Figure S4). Furthermore, the activities of CAT and POD were enhanced in the *RcHsf17* overexpression transgenic lines under 42 °C conditions (Figure 9). These results show that *RcHsf17* overexpression improves thermotolerance in transgenic *Arabidopsis* by regulating the expression changes of *AtHsps* and increasing the activities of antioxidant enzymes.

### 2.8. RcHsf17 Enhances Sensitivity to MeJA in Transgenic Arabidopsis

**Figure 10:**
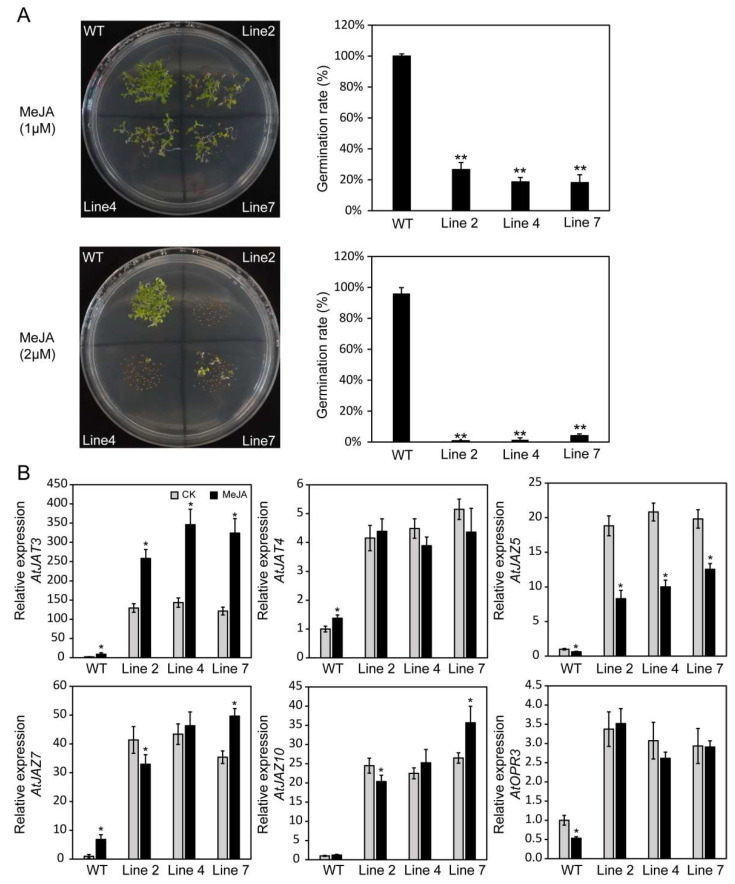
MeJA stress response of overexpressing *RcHsf17* lines. (**A**) The phenotypes and germination rate of overexpressing *RcHsf17* lines and WT seedlings grown on 1/2 MS medium supplemented with 1 μM and 2 μM of methyl jasmonate; (**B**) The expression of JA synthesis and regulation-related genes *AtJAT3*, *AtJAT4*, *AtJAZ5*, *AtJAZ7*, *AtJAZ10*, and *AtOPR3*. Data are means ± SD of three biological replicates. The asterisks on the top of bars indicate significant differences between *RcHsf17* overexpression transgenic lines and wild type. * *p* < 0.05, ** *p* < 0.01 by *t*-test.

Based on the cis-element analysis of the *RcHsf17* promoter sequence and the expression profile of *RcHsf17* under MeJA treatment, we speculated that *RcHsf17* might play a crucial role in the JA-dependent defense. To verify this speculation, we treated wild-type and transgenic lines with 1 μM and 2 μM of MeJA. We found that the *RcHsf17* overexpression transgenic lines grew slowly after treatment with 1 μM of MeJA, and the germination rate was significantly lower than that of the wild type. When the MeJA concentration was set to 2 μM of MeJA, transgenic line 2 was unable to germinate normally (Figure 10A). The overexpression of *RcHsf17* significantly increased the expression of jasmonic acid synthesis and regulation-related genes such as *AtJAT3*, *AtJAT4*, *AtJAZ5*, *AtJAZ7*, *AtJAZ10*, and *AtOPR3* (Figure 10B). When treated with 1 μM of MeJA, the expression of *AtJAT3* in the transgenic lines significantly increased compared with the wild type, while the expression of *AtJAZ5* significantly decreased (Figure 10B). Therefore, we speculated that the overexpression of *RcHsf17* could enhance the sensitivity of *Arabidopsis* to MeJA, thereby inhibiting the germination of *Arabidopsis* seeds.

## 3. Discussion

Plant heat shock transcription factors play vital roles in the response to high-temperature stress. There are 19 *Hsf* genes in roses, which is less than in most other plant species (Figure 1). The number of *Hsf* genes in roses and grapes is the same, and their genome size (515 Mb:495 Mb) and number of genes (36,377:37,534) are similar [23,24]. Moreover, similar to other species, the 19 *RcHsfs* are divided into three main subfamilies (A, B, and C). Subfamily A Hsfs form the largest subfamily with 12 members, followed by subfamily B with 6 *RcHsf* genes. Subfamily C *Hsfs* only have one member, *RcHsf3* (Figure 2A). Although the subfamilies A, B, and C are generally similar in structure, there are still some differences in specific domains. For example, *RcHsf12* lacks motif 3 (DBD domain) in subfamily B, which is an important component motif of the typical domain of the *Hsf* gene family (Figure 2B). In addition, *RcHsf3* (subfamily C) has the AHA motif, which is reported to only be located in all subfamily A *Hsfs* [25] (Figure 2B). We speculate that a possible motif loss and the presence of *RcHsfs* in the evolution process may result in the specific regulation and functional pattern of these genes.

The number of *Hsf* genes is the most abundant in subfamily A in plants, including in roses. Compared with subfamily A *Hsfs*, subfamily B and subfamily C *Hsfs* are generally considered repressive *Hsfs* [11,12,26]. In roses, the subfamily B *Hsf* genes *RcHsf2, RcHsf5*, and *RcHsf18* can be induced by high temperature. The mutation of *S. lycopersicum*, SlHsfB1, can also cause changes in genes related to the high-temperature response [27]. There are few studies on the function of subfamily C members [28]. In the current study, there was only one subfamily C *Hsf* gene (*RcHsf3*) in the rose genome, and its expression decreased at high temperatures. Interestingly, the number of motifs in the *RcHsf3* promoter sequence was the largest, most of which were light-responsive elements and hormone-responsive elements (Figure 5).

*Cis*-acting elements are considered to be related to gene initiation and transcription and are widely found in promoters and are conserved within species [29,30]. In *Arabidopsis, HsfA6b* was directly bound to the promoter of *AtDREB2A* and enhanced its expression to participate in ABA-mediated salt and drought resistance. We found four mainly regulatory motifs in *RcHsf* gene promoters, and the distribution of these motifs in each *Hsf* gene was significantly different (Figure 3A). Consistent with other species, the stress-responsive elements in most *RcHsf* promoters were detected, which is consistent with the important function of *RcHsfs* in response to stress [31]. In addition, hormone-responsive elements were also widely distributed in the *RcHsf* promoters (Figure 3B). Among them, the abscisic acid response element (ABRE) was the most abundant, with 37, and was widely distributed in 16 *RcHsfs* promoters (Figure 3B).

Plants respond to high temperatures by regulating the expression of related genes. However, not all high temperatures can induce the expression of *Hsf* genes. Moderate elevated temperatures will trigger the expression changes of genes related to plant thermomorphogenesis to adapt to temperature changes [1]. We found that *RcHsf* genes had different expression patterns when exposed to different high-temperature conditions. At a warm temperature (30 °C), the expression of nine *RcHsf* genes increased, including five subfamily B (*RcHsf2*, *RcHsf8*, *RcHsf12*, *RcHsf16*, and *RcHsf18*) and four subfamily A (*RcHsf1*, *RcHsf4*, *RcHsf15*, and *RcHsf17*) *RcHsf* genes (Figure 5). However, at 42 °C, there were 12 *RcHsf* genes from subfamily A (*RcHsf1*, *RcHsf4*, *RcHsf9*, *RcHsf10*, *RcHsf13*, *RcHsf15*, and *RcHsf17*) and subfamily B (*RcHsf2*, *RcHsf5*, *RcHsf12*, *RcHsf16*, and *RcHsf18)* that could be significantly up-regulated (Figure 5). In all Hsf genes, the expression of *RcHsf3, RcHsf6, RcHsf11, RcHsf14*, and *RcHsf19* could not be induced by high temperatures from 30 °C to 42 °C, and even the expression was significantly reduced. So, we speculated that these *RcHsfs* may be located downstream of the high temperature or may be inhibited by other *Hsf* genes. This expression pattern has also been verified in other *Hsf* family genes [5]. The different expression profiles of *RcHsf* genes might be related to different functions in response to abiotic stresses.

*AtHsfA3* transcript levels increased at high temperatures in *Arabidopsis*, and the antioxidant gene *AtAPX2* was up-regulated, thereby improving oxidative stress tolerance in *AtHsfA3* overexpression plants. In addition, the *HsfA3* mutant showed substantially reduced thermotolerance [32]. *AtHsfA3* overexpression enhances *Arabidopsis* heat resistance but may increase susceptibility to salt [33] In *Zea mays*, *ZmHsfA2* overexpression enhanced plant thermotolerance by increasing the expression of *Arabidopsis* galactinol synthase genes *AtGOLS1* and *AtGOLS2* and raffinose synthase gene *AtRS5* and then increasing the raffinose content in leaves [34]. *HvHsfA6a* overexpression plants show lower ROS accumulation and enhanced antioxidative potential under high-temperature conditions [20]. Our results show that *RcHsf17* was significantly induced at high temperatures, and its overexpression in *Arabidopsis* increased the expression of the antioxidant gene *AtAPX2* and the activities of CAT and POD, and then thermotolerance increased (Figure 8 and Figure 9). These Hsf gene functions are relatively conservative, but they have certain specificity in different plants.

*Hsf* transmits high-temperature information and improves plant stress resistance mainly by regulating the expression of *Hsp* genes [35,36]. The transcript levels of *Hsp18.1, AtHsp22*, *AtHsp25*, and *AtAPX2* were substantially affected by high temperatures in an *Arabidopsis HsfA2* mutant [12]. In wheat, *TaHsfA6e* directly binds to *TaHsp70s* promoters and up-regulates its expression in response to high temperatures [37]. *AtHsp101* is essential for survival at severe high temperatures and for the acquisition of thermotolerance [38,39]. In the current study, we found *RcHsf17* overexpression improved the thermotolerance of *Arabidopsis* by promoting the expression of *Hsp* genes and stress-related genes, such as *AtHsp18*, *AtHsp22*, *AtHsp25*, *AtHsp26*, *AtHsp70*, and *AtAPX2*. Under 42 °C conditions, the expression of *AtHsp18*, *AtHsp22*, *AtHsp25*, *AtHsp26*, *AtHsp70*, and *AtAPX2* in *RcHsf17* overexpression lines was significantly higher than that in wild-type lines. Based on this, we hypothesized that *Hsp18*, *Hsp22*, *Hsp25*, *Hsp26*, and *AtHsp70* may be targets of *RcHsf17* and participate in improving the thermotolerance of *Arabidopsis*. However, the regulatory network still needs to be further explored. Previous studies have found that *AtHsfA2* and *OsHsfA2* can enhance salt tolerance in transgenic *Arabidopsis* [6,40].

MeJA plays an important role in plant development and defense against abiotic stressors [41]. Low concentrations of MeJA can induce JA synthesis, enhance the expression of defense-related genes, and alleviate stress-induced damage. However, high concentrations of MeJA inhibit seed or pollen germination [42]. Notably, when HsfA1d is overexpressed in cucumbers, it can trigger the biosynthesis and signal transmission of endogenous jasmonic acid (JA), resulting in enhanced cold tolerance in cucumbers [43]. MeJA treatment decreases the germination rate of the seeds of RcHsf17 overexpression *Arabidopsis* and increases the expression of genes associated with JA synthesis (Figure 10), in which *AtJAT3* and *AtJAZ5* show a completely opposite expression pattern. *RcHsf17* overexpression reduces *AtJAZ5* expression, and the degradation of the JAZ protein is a key step in uninhibiting the JA pathway [44]. Recent studies have shown that RhJAZ5 interacts with RhCIPK3 to regulate petal senescence in roses [45]. We therefore speculate that the heterologous expression of *RcHsf17* affects seed germination by attenuating the expression of endogenous JA synthesis genes in *Arabidopsis*.

As a basic transcription factor family that plays an important role in high-temperature stress, the molecular mechanism of *Hsf* genes in the heat tolerance of roses provides an important reference. *RcHsf17* was induced and achieved the highest expression at high temperatures. The overexpression of *RcHsf17* increased thermotolerance in transgenic *Arabidopsis*. Generally, the results of this study provide new insights into the molecular mechanisms of *RcHsfs.*

## 4. Materials and Methods

### 4.1. Identification of Hsf Gene Families in R. chinensis

The amino acid sequences of *R.chinensis* were downloaded from RchiOBHm-V2 (https://lipm-browsers.toulouse.inra.fr/pub/RchiOBHm-V2) (accessed on 15 March 2024) [23]. The amino acid sequences of *Hsfs* from *Arabidopsis* and *S*. *lycopersicum* were downloaded from the TAIR database (https://www.arabidopsis.org/) (accessed on 15 March 2024) [46] and SGN database (https://www.solgenomics.net/) (accessed on 15 March 2024) [47], respectively (Tables S1 and S2). To identify all possible *RcHsfs*, the Hsf-type DBD domain (Pfam: PF00447) was searched against the *R. chinensis* proteome using the HMMER3 tool (version 3.1), and the E value was set to E < 10^−3^ [48,49]. ExPASy (http://www.expasy.org/tools/) (accessed on 14 June 2024) was used for calculating the molecular weight (kDa) and isoelectric point (pI) of the *RcHsfs* [50].

### 4.2. Chromosomal Location and Phylogenetic Analysis of RcHsfs

The chromosome location information of the *RcHsfs* came from RchiOBHm-V2, including chromosome length and chromosome location. TBtools was used to draw a chromosome location map [51]. All the *RcHsfs* were predicted via Clustal-W software (https://www.genome.jp/tools-bin/clustalw) (accessed on 14 July 2024), and the phylogenetic relationships were analyzed by the MEGA 5.1 program and the neighbor-joining method according to the multiple sequence alignments with orthologs from *Arabidopsis* and tomatoes (*S. lycopersicum*) [52]. Finally, the phylogenetic tree was designed by the online tool ITOL (https://itol.embl.de/) (accessed on 14 July 2024) [53].

### 4.3. Cis-Acting Element Analysis of the RcHsf Promoters

The promoter sequence was derived from the 2000 bp upstream sequence of the 19 *Hsf* genes in the *R. chinensis* genome database. The detailed sequence information is shown in Table S3. The *cis*-acting elements were predicted using the PlantCARE database (http://bioinformatics.psb.ugent.be/webtools/plantcare/html/) (accessed on 14 July 2024) [54].

### 4.4. The Structural and Motif Analysis of RcHsfs

The exon/intron structures of the *RcHsfs* from RchiOBHm-V2 were analyzed by comparing their cDNA sequences and genomic DNA sequences. The structures map of the *RcHsfs* was drawn using TBtools (version 2.056) [51]. The conserved motifs of the RcHsfs were analyzed by the MEME program (http://meme-suite.org/tools/meme) (accessed on 20 October 2024). The number of repetitions was set to any, and the maximum number of motifs was set to 10. The optimal motif length was between 6 and 50 residues [55].

### 4.5. Plant Materials and Stress Treatment

Chinese ancient roses (*R. chinensis* ‘Semperflorens’ cv. ‘Slater’s Crimson China’) are a good material for studying the heat resistance of roses because of their flowering characteristics over four seasons. They were cultivated in a greenhouse at the Institute of Forestry and Pomology, Beijing Academy of Agriculture and Forestry Sciences, Beijing, China. Three-year-old potted *R. chinensis* seedlings, in which the flower traits are relatively stable, were used as experimental materials. The flower development process was from stage 1 (S1) to stage 5 (S5). S1 is the small bud stage with the bud closed. S2 is the big bud stage with the petals slightly exposed. S3 is the initial opening stage with the petals extended. S4 is the full blooming stage with the petals fully open. S5 is the late blooming stage with curly petals [56]. Petals from the five flower development stages were sampled to analyze gene expression. The roots, stems, leaves, and petals of the roses were sampled from seedlings at the full bloom stage. The samples were quickly frozen in liquid nitrogen and stored at −80 °C until further use.

Seedlings at the bud stage were transferred in an artificial climate box, the parameters of which were set to a photoperiod of 16/8 h light/darkness, 4800 LX light, and 60% relative humidity. Until the full bloom stage, the seedlings were used for high-temperature treatment. The processing temperature was set to the normal temperature of 25 °C and high temperatures of 30 °C, 35 °C, 37 °C, and 42 °C. The petals at different temperatures were sampled after the high-temperature treatment for 0 h, 15 min, 30 min, and 1 h, respectively. The samples were quickly frozen using liquid nitrogen and stored at −80 °C until further use.

### 4.6. Real-Time Quantitative PCR (RT-PCR) Assay of the RcHsf Genes

Total RNA was extracted with an RNA isolation kit (Tiangen Biotech Co., Beijing, China). The spectrophotometer was utilized to determine the concentration of RNA, while gel electrophoresis was employed to assess its integrity. The cDNA was produced utilizing a first strand cDNA synthesis kit (TransGen Biotech, Beijing, China). Subsequently, qPCR amplifications were performed with a Bio-Rad CFX96 PCR system using TB Green^®^ Premix Ex Taq™ (Tli RNase H Plus) (Takara, Shiga, Japan). The reaction procedure followed the manufacture’s recommended protocol [27]. The sequences of primers are listed in Table S4. *R. chinensis* actin (GenBank accession: KC514920) was used as the internal control. The 2^−∆∆Ct^ method was used to calculate the relative expression ratio. The RT-PCR analysis was performed with three technical replicates. Data are presented as the mean ± SE [57].

### 4.7. Transactivation Activity Assay

The full-length coding sequence of *RcHsf17* (GenBank accession: PP622669, unreleased) was fused in-frame to the pGBKT7 vector via SalI and NdeI restriction sites by seamless cloning. The fusion constructs were transferred into Y187 yeast cells (WEIDI, YC1020) according to the protocol and then coated on a SD/-Trp solid medium. After 48~72 h of growth at 30 °C, four yeast cells fused with pGBKT7-*RcHsf17* were transferred to a SD/-Trp solid medium and SD/-Trp medium containing X-α-gal. The growth state of the yeast cells was observed and photographed after 48~72 h. The positive and negative controls were pGBKT7-p53 and pGBKT7 plasmids, respectively [58].

### 4.8. Prediction of Subcellular Localization and the Subcellular Localization Analysis of RcHsf17

The subcellular localization of the 19 RcHsfs was predicted by the online tool Plant-mPLoc (http://www.csbio.sjtu.edu.cn/bioinf/plant/) (accessed on 25 October 2024) [59]. The recombinant plasmid super1300-RcHsf17-GFP (*35S*::RcHsf17) was constructed via SpeI and KpnI restriction sites by seamless cloning (Figure S2). Three-week-old *Arabidopsis* seedlings grown in an artificial climate box, the parameters of which were set to 23 °C, a photoperiod of 16/8 h light/darkness, 4800 LX light, and 60% relative humidity, were used to extract protoplasts. Then, 10 μL (1000 ng/μL) of recombinant plasmids *35S*::RcHsf17 and super1300-GFP (*35S*::GFP) (negative control) were transformed into *Arabidopsis* protoplasts. The extraction and transformation of the *Arabidopsis* protoplasts were carried out using an *Arabidopsis* protoplast preparation and transformation kit (Coolaber, PPT101) according to the manufacturer’s instructions. After incubation for 12 h, the fluorescence was observed under a confocal laser scanning microscope (Nikon A1R, Tokyo, Japan). GFP was determined at the excitation wavelength of 488 nm and the emission wavelength of 507 nm; RFP was determined at the excitation wavelength of 532 nm and the emission wavelength of 588 nm. H_2_B-mCherry was used as a positive marker [60].

### 4.9. Western Blot

Proteins were extracted from the *Arabidopsis* seedlings using a plant protein extraction kit (Toyephon, Beijing, China). The Western blot experiments were carried out as described by Kang [61]. Briefly, 12% sodium dodecyl sulfate-polyacrylamide gel, nitrocellulose membranes, anti-GFP rabbit polyclonal antibody, and goat anti-rabbit polyclonal antibody were purchased from Beyotime (Shanghai, China). The membranes were incubated with the anti-GFP rabbit polyclonal antibody and goat anti-rabbit polyclonal antibody successively and then visualized using the Immobilon Western Chemiluminescent HRP substrate (Millipore Corp., Billerica, MA, USA). All analyses were repeated three times.

### 4.10. Generation of RcHsf17 Overexpression Transgenic Arabidopsis

The recombinant plasmid *35S*::RcHsf17 was transformed into *Arabidopsis* using the classic floral dip method at the stage of flower budding [62]. The *RcHsf17* overexpression transgenic *Arabidopsis* seeds were selected on an MS medium supplemented with 50 mg/L of hygromycin and verified by PCR and Western blot. The identified homozygous transgenic seeds were used for further processing.

### 4.11. High-Temperature Treatment of RcHsf17 Overexpression Transgenic Arabidopsis

For the high-temperature treatment, the vernalized seeds of the wild-type and homozygous transgenic lines were sterilized using 75% alcohol for 1 min and then rinsed with sterile water four times. The vernalized seeds of the wild-type and homozygous transgenic lines were planted on MS agar plates, which are more conducive to the statistical germination rate and root hair phenotype. Plates of wild-type and transgenic lines were placed in dark conditions at 4 °C for 2 d and then transferred to a 23 °C artificial climate box for 14 d, the parameters of which were set to a photoperiod of 16/8 h light/darkness, 4800 LX light, and 60% relative humidity. Then, the seedlings of the wild-type and transgenic lines were placed at 42 °C for 2 h, and the survival rates were calculated after a 14-day recovery at 22 °C. Moreover, the wild-type and transgenic seedlings were transferred to soil for 15 days, the phenotype was observed, and samples were collected, and a transient temperature treatment experiment at 42 °C for 15 min, 30 min, and 1 h was carried out. At the same time, samples were collected for RT-PCR [57].

### 4.12. Methyl Jasmonate (MeJA) Treatment

For the MeJA treatment, the sterilized seeds of the wild-type and transgenic lines were planted on MS agar plates containing 1 μm of MeJA and 2 μm of MeJA. After 14 days, the phenotype was observed, and the germination rate was determined. At the same time, samples were collected for real-time quantitative PCR detection.

### 4.13. Statistical Analysis

The qRT-PCR data were analyzed by IBM SPSS Statistics 26 (IBM Corp., Armonk, NY, USA). Bivariate correlation analysis was used to detect the correlation of the gene transcription levels between the normal and high-temperature treatment and the correlation of the gene transcription levels between the wild-type and *RcHsf17* overexpression transgenic lines. Each experiment was repeated three times.

## 5. Conclusions

In this study, we identified Hsf family genes from *R. Chinensis* and analyzed the characteristics and expression patterns of RcHsfs. Among the 19 RcHsfs, *RcHsf17* had the highest expression during the high-temperature treatment. The heterologous expression of *RcHsf17* increased the heat stress tolerance in transgenic *Arabidopsis*, suggesting its potential role in the regulation of the heat stress response. Collectively, this study identified and screened RcHsf responsiveness to high temperatures in roses, providing new insights into the functional divergence of RcHsfs and a basis for further research on RcHsf functions.

## Figures and Tables

**Table 1:** Basic information of *Hsf* family genes in *R. chinensis*.

Gene Name	Gene ID	Accession Number	CDS (bp)	Peptide (aa)	Predicted Isoelectric (PI)	Molecular Weight (kD)	Chr	Type	Predicted Localization
*RcHsf1*	XP_024163952.1	Unnamed	564	187	5.48	28,368.21	1	A	Nucleus
*RcHsf2*	XP_024169367.1	Chr1g0376961	870	289	8.49	32,202.24	1	B	Nucleus
*RcHsf3*	XP_024181902.1	Chr2g0090441	1089	362	6.13	40,611.18	2	C	Nucleus
*RcHsf4*	XP_024184386.1	Chr2g0093671	1404	467	5.19	52,332.68	2	A	Nucleus
*RcHsf5*	XP_024181588.1	Chr2g0105601	894	297	6.18	32,701.44	2	B	Nucleus
*RcHsf6*	XP_024181332.1	Chr2g0131371	1314	437	4.98	49,595.16	2	A	Nucleus
*RcHsf7*	XP_024187370.1	Chr3g0472141	1155	384	4.51	14,478.46	3	A	Nucleus
*RcHsf8*	XP_024189461.1	Chr3g0482741	726	241	6.06	27,641.04	3	B	Nucleus
*RcHsf9*	XP_024197380.1	Chr4g0387751	1053	350	5.33	40,432.6	4	A	Nucleus
*RcHsf10*	XP_024193341.1	Chr4g0413481	1431	476	5.49	53,177.84	4	A	Nucleus
*RcHsf11*	XP_024193980.1	Chr4g0442621	1467	488	4.71	55,655.11	4	A	Nucleus
*RcHsf12*	XP_024159032.1	Chr5g0008251	387	128	5.1	14,706.41	5	B	Nucleus
*RcHsf13*	XP_024157545.1	Chr5g0011961	1311	436	5.18	48,895.43	5	A	Nucleus
*RcHsf14*	XP_024158000.1	Chr5g0027071	1479	492	5.3	55,084.97	5	A	Nucleus
*RcHsf15*	XP_024165207.1	Chr6g0290041	927	308	9.21	35,192.12	6	A	Nucleus
*RcHsf16*	XP_024166365.1	Chr6g0308161	1188	395	8.14	44,263.68	6	B	Nucleus
*RcHsf17*	XP_024168362.1	Chr6g0311151	1128	375	4.88	41,853.78	6	A	Nucleus
*RcHsf18*	XP_024174102.1	Chr7g0198941	1041	346	5.03	37,887.21	7	B	Nucleus
*RcHsf19*	XP_024170740.1	Chr7g0211291	1578	525	4.79	57,118.38	7	A	Nucleus

## Data Availability

The original contributions presented in this study are included in the article/Supplementary Material. Further inquiries can be directed to the corresponding author.

## Supplementary Materials

The following supporting information can be downloaded at: https://www.mdpi.com/article/10.3390/ijms26010287/s1.

## References

[1] Kan Y., Mu X., Gao J., Lin H., Lin Y. (2023). The molecular basis of heat stress responsesin plants. Mol. Plant..

[2] de Vries D.P., Dubois L.A.M. (1996). Rose breeding past, present, prospects. Acta Hortic..

[3] Zhu Q.Y., Zhang L.L., Liu J.X. (2024). NFXL1 functions as a transcriptional activator required for thermotolerance at reproductive stage in *Arabidopsis*. J. Integr. Plant Biol..

[4] Ma Z., Li M., Zhang H., Zhao B., Liu Z., Duan S., Meng X., Li G., Guo X. (2023). Alternative Splicing of *TaHsfA2-7* Is Involved in the Improvement of Thermotolerance in Wheat. Int. J. Mol. Sci..

[5] Rao S., Gupta A., Bansal C., Sorin C., Crespi M., Mathur S. (2022). A conserved HSF miR169 NF-YA loop involved in tomato and *Arabidopsis* heat stress tolerance. Plant J..

[6] Friedrich T., Oberkofler V., Trindade I., Altmann S., Brzezinka K., Lamke J., Gorka M., Kappel C., Sokolowska E., Skirycz A. (2021). Heteromeric HSFA2/HSFA3 complexes drive transcriptional memory after heat stress in *Arabidopsis*. Nat. Commun..

[7] Yang X., Zhu W., Zhang H., Liu N., Tian S. (2016). Heat shock factors in tomatoes: Genome-wide identification, phylogenetic analysis and expression profiling under development and heat stress. PeerJ.

[8] Fu J., Huang S., Qian J., Qing H., Wan Z., Cheng H., Zhang C. (2022). Genome-Wide Identification of Petunia HSF Genes and Potential Function of PhHSF19 in Benzenoid/Phenylpropanoid Biosynthesis. Int. J. Mol. Sci..

[9] Song X., Liu G., Duan W., Liu T., Huang Z., Ren J., Li Y., Hou X. (2014). Genome-wide identification, classification and expression analysis of the heat shock transcription factor family in Chinese cabbage. Mol. Genet. Genom. MGG.

[10] Nakai A. (2016). Molecular basis of HSF regulation. Nat. Struct. Mol. Biol..

[11] Iqbal M.Z., Jia T., Tang T., Anwar M., Ali A., Hassan M.J., Zhang Y., Tang Q., Peng Y. (2022). A Heat Shock Transcription Factor TrHSFB2a of White Clover Negatively Regulates Drought, Heat and Salt Stress Tolerance in Transgenic *Arabidopsis*. Int. J. Mol. Sci..

[12] Liu X., Chen H., Li S., Lecourieux D., Duan W., Fan P., Liang Z., Wang L. (2023). Natural variations of HSFA2 enhance thermotolerance in grapevine. Hortic. Res.-Engl..

[13] Lin K.F., Tsai M.Y., Lu C.A., Wu S.J., Yeh C.H. (2018). The roles of *Arabidopsis* HSFA2, HSFA4a, and HSFA7a in the heat shock response and cytosolic protein response. Bot. Stud..

[14] Liu B., Hu J., Zhang J. (2019). Evolutionary Divergence of Duplicated Hsf Genes in Populus. Cells.

[15] Kerbler S.M., Wigge P.A. (2023). Temperature Sensing in Plants. Annu. Rev. Plant Biol..

[16] Fragkostefanakis S., Mesihovic A., Simm S., Paupière M.J., Hu Y.P.P., Mishra S.K., Tschiersch B., Theres K.B.A., Schleiff E., Scharf K.D. (2016). HsfA2 Controls the Activity of Developmentally and Stress-Regulated Heat Stress Protection Mechanisms in Tomato Male Reproductive Tissues. Plant Physiol..

[17] Wang N., Liu W., Yu L., Guo Z., Chen Z., Jiang S., Xu H., Fang H., Wang Y., Zhang Z. (2020). HEAT SHOCK FACTOR A8a Modulates Flavonoid Synthesis and Drought Tolerance. Plant Physiol..

[18] Li Z., Tang J., Srivastava R., Bassham D.C., Howell S.H. (2020). The Transcription Factor bZIP60 Links the Unfolded Protein Response (UPR) to the Heat Stress Response (HSR) in Maize. Plant Cell.

[19] Wu Z., Li T., Ding L., Wang C., Teng R., Xu S., Cao X., Teng N. (2024). Lily LlHSFC2 coordinates with HSFAs to balance heat stress response and improve thermotolerance. New Phytol..

[20] Chaudhary R., Baliyan S., Sirohi P., Singh S., Mishra S.K., Rajkumar M.S., Saini S.S., Germain H., Sircar D., Chauhan H. (2024). Overexpression of barley heat stress transcription factor HvHsfA6a provide thermotolerance by thermopriming. bioRxiv.

[21] Andrási N., Pettkó-Szandtner A., Szabados L. (2021). Diversity of plant heat shock factors regulation, interactions, and functions. J. Exp. Bot..

[22] Ding X., Guo Q., Li Q., Gai J., Yang S. (2020). Comparative Transcriptomics Analysis and Functional Study Reveal Important Role of High-Temperature Stress Response Gene GmHSFA2 During Flower Bud Development of CMS-Based F1 in Soybean. Front. Plant Sci..

[23] Raymond O., Gouzy J., Just J., Badouin H., Verdenaud M., Lemainque A., Vergne P., Moja S., Choisne N., Pont C. (2018). The Rosa genome provides new insights into the domestication of modern roses. Nat. Genet..

[24] Liang Z., Duan S., Sheng J., Zhu S., Ni X., Shao J., Liu C., Nick P., Du F., Fan P. (2019). Whole-genome resequencing of 472 Vitis accessions for grapevine diversity and demographic history analyses. Nat. Commun..

[25] Guo J., Jian Wu Q.J., Wang C., Luo L., Yuan Y., Wang Y., Wang J. (2008). Genome-wide analysis of heat shock transcription factor families in rice and *Arabidopsis*. J. Genet. Genom..

[26] Zhang Y., Wang C., Wang C., Yun L., Song L., Idrees M., Liu H., Zhang Q., Yang J., Zheng X. (2022). OsHsfB4b Confers Enhanced Drought Tolerance in Transgenic *Arabidopsis* and Rice. Int. J. Mol. Sci..

[27] Fragkostefanakis S., Simm S., El-Shershaby A., Hu Y., Bublak D., Mesihovic A., Darm K., Mishra S.K., Tschiersch B., Theres K. (2019). The repressor and co-activator HsfB1 regulates the major heat stress transcription factors in tomato. Plant Cell Environ..

[28] Zhuang L., Cao W., Wang J., Yu J., Yang Z., Huang B. (2018). Characterization and Functional Analysis of FaHsfC1b from Festuca arundinacea Conferring Heat Tolerance in *Arabidopsis*. Int. J. Mol. Sci..

[29] Chen M., He X., Huang X., Lu T., Zhang Y., Zhu J., Yu H., Luo C. (2022). Cis-element amplified polymorphism (CEAP), a novel promoter- and gene-targeted molecular marker of plants. Physiol. Mol. Biol. Plants.

[30] Bennetzen J.L., Wang X. (2018). Relationships between Gene Structure and Genome Instability in Flowering Plants. Mol. Plant..

[31] Zhang X., Xu W., Ni D., Wang M., Guo G. (2020). Genome-wide characterization of tea plant (*Camellia sinensis*) Hsf transcription factor family and role of CsHsfA2 in heat tolerance. Bmc Plant Biol..

[32] Song C., Chung W.S., Lim C.O. (2016). Overexpression of Heat Shock Factor Gene *HsfA3* Increases Galactinol Levels and Oxidative Stress Tolerance in *Arabidopsis*. Mol. Cells.

[33] Wu Z., Liang J., Wang C., Zhao X., Zhong X., Cao X., Li G., He J., Yi M. (2018). Overexpression of lily HsfA3s in *Arabidopsis* confers increased thermotolerance and salt sensitivity via alterations in proline catabolism. J. Exp. Bot..

[34] Gu L., Jiang T., Zhang C., Li X., Wang C., Zhang Y., Li T., Dirk L.M.A., Downie A.B., Zhao T. (2019). Maize HSFA2 and HSBP2 antagonistically modulate raffinose biosynthesis and heat tolerance in *Arabidopsis*. Plant J. Cell Mol. Biol..

[35] Gorovits R., Shteinberg M., Anfoka G., Czosnek H. (2022). Exploiting Virus Infection to Protect Plants from Abiotic Stresses: Tomato Protection by a Begomovirus. Plants.

[36] Song Q., He F., Kong L., Yang J., Wang X., Zhao Z., Zhang Y., Xu C., Fan C., Luo K. (2023). The IAA17.1/HSFA5a module enhances salt tolerance in Populus tomentosa by regulating flavonol biosynthesis and ROS levels in lateral roots. New Phytol..

[37] Wen J., Qin Z., Sun L., Zhang Y., Wang D., Peng H., Yao Y., Hu Z., Ni Z., Sun Q. (2023). Alternative splicing of TaHSFA6e modulates heat shock protein-mediated translational regulation in response to heat stress in wheat. New Phytol..

[38] Katiyar-Agarwal S., Grover M.A.A.A. (2003). Heat-tolerant basmati rice engineered by over-expression of *hsp101*. Plant Mol.Biol..

[39] Qin F., Yu B., Li W. (2021). Heat shock protein 101 (HSP101) promotes flowering under nonstress conditions. Plant Physiol..

[40] Charng Y., Liu H., Liu N., Chi W., Wang C., Chang S., Wang T. (2007). A Heat-Inducible Transcription Factor, HsfA2, Is Required for Extension of Acquired Thermotolerance in *Arabidopsis*. Plant Physiol..

[41] Nahar K., Kyndt T., De Vleesschauwer D., Höfte M., Gheysen G. (2011). The Jasmonate Pathway Is a Key Player in Systemically Induced Defense against Root Knot Nematodes in Rice. Plant Physiol..

[42] Han X., Zhang M., Yang M., Hu Y. (2020). *Arabidopsis* JAZ Proteins Interact with and Suppress RHD6 Transcription Factor to Regulate Jasmonate-Stimulated Root Hair Development. Plant Cell.

[43] Qi C., Dong D., Li Y., Wang X., Guo L., Liu L., Dong X., Li X., Yuan X., Ren S. (2022). Heat shock-induced cold acclimation in cucumber through CsHSFA1d-activated JA biosynthesis and signaling. Plant J. Cell Mol. Biol..

[44] Li M., Wang F., Li S., Yu G., Wang L., Li Q., Zhu X., Li Z., Yuan L., Liu P. (2020). Importers Drive Leaf-to-Leaf Jasmonic Acid Transmission in Wound-Induced Systemic Immunity. Mol. Plant..

[45] Chen C., Ma Y., Zuo L., Xiao Y., Jiang Y., Gao J. (2023). The CALCINEURIN B-LIKE 4/CBL-INTERACTING PROTEIN 3 module degrades repressor JAZ5 during rose petal senescence. Plant Physiol..

[46] Rocha J.J., Jayaram S.A., Stevens T.J., Muschalik N., Shah R.D., Emran S., Robles C., Freeman M., Munro S., Dunham I. (2023). Functional unknomics: Systematic screening of conserved genes of unknown function. Plos. Biol..

[47] Fernandez-Pozo N., Menda N., Edwards J.D., Saha S., Tecle I.Y., Strickler S.R., Bombarely A., Fisher-York T., Pujar A., Foerster H. (2015). The Sol Genomics Network (SGN)--from genotype to phenotype to breeding. Nucleic Acids Res..

[48] Finn R.D., Coggill P., Eberhardt R.Y., Eddy S.R., Mistry J., Mitchell A.L., Potter S.C., Punta M., Qureshi M., Sangrador-Vegas A. (2016). The Pfam protein families database: Towards a more sustainable future. Nucleic Acids Res..

[49] Eddy S.R. (2011). Accelerated Profile HMM Searches. PLoS Comput. Biol..

[50] Ison J., Kalas M., Jonassen I., Bolser D., Uludag M., Mcwilliam H., Malone J., Lopez R., Pettifer S., Rice P. (2013). EDAM: An ontology of bioinformatics operations, types of data and identifiers, topics and formats. Bioinformatics.

[51] Chen C., Chen H., Zhang Y., Thomas H.R., Frank M.H., He Y., Xia R. (2020). TBtools: An Integrative Toolkit Developed for Interactive Analyses of Big Biological Data. Mol. Plant..

[52] Tamura K., Peterson D., Peterson N., Stecher G., Nei M., Kumar S. (2011). MEGA5: Molecular evolutionary genetics analysis using maximum likelihood, evolutionary distance, and maximum parsimony methods. Mol. Biol. Evol..

[53] Letunic I., Bork P. (2024). Interactive Tree of Life (iTOL) v6: Recent updates to the phylogenetic tree display and annotation tool. Nucleic Acids Res..

[54] Lescot M., Déhais P., Thijs G., Marchal K., Moreau Y., Van de Peer Y., Rouzé P., Rombauts S. (2002). PlantCARE, a database of plant cis-acting regulatory elements and a portal to tools for in silico analysis of promoter sequences. Nucleic Acids Res..

[55] Bailey T.L., Boden M., Buske F.A., Frith M., Grant C.E., Clementi L., Ren J., Li W.W., Noble W.S. (2009). MEME SUITE: Tools for motif discovery and searching. Nucleic Acids Res..

[56] Guterman I., Shalit M., Menda N., Piestun D., Dafny-Yelin M., Shalev G., Bar E., Davydov O., Ovadis M., Emanuel M. (2002). Rose Scent: Genomics Approach to Discovering Novel Floral Fragrance-Related Genes. Plant Cell.

[57] Livak K.J., Schmittgen T.D. (2001). Analysis of Relative Gene Expression Data Using Real-Time Quantitative PCR and the 2^−ΔΔCT^ Method. Methods.

[58] Gong Z., Luo Y., Zhang W., Jian W., Zhang L., Gao X., Hu X., Yuan Y., Wu M., Xu X. (2021). A SlMYB75-centred transcriptional cascade regulates trichome formation and sesquiterpene accumulation in tomato. J. Exp. Bot..

[59] Chou K., Shen H. (2008). Cell-PLoc: A package of Web servers for predicting subcellular localization of proteins in various organisms. Nat. Protoc..

[60] Ouyang M., Yu J.Y., Chen Y., Deng L., Guo C.L. (2021). Cell-extracellular matrix interactions in the fluidic phase direct the topology and polarity of self-organized epithelial structures. Cell Prolif..

[61] Kang Y., Li W., Zhang L., Qi L. (2021). Over-Expression of the Cell-Cycle Gene *LaCDKB1;2* Promotes Cell Proliferation and the Formation of Normal Cotyledonary Embryos during Larix kaempferi Somatic Embryogenesis. Genes..

[62] Clough S.J., Bent A.F. (1998). Floral dip: A simplified method for Agrobacterium-mediated transformation of *Arabidopsis thaliana*. Plant J. Cell Mol. Biol..

