# Supplementary material for: Genome-Wide Analysis of the Hsf Gene Family in Rosa chinensis and RcHsf17 Function in Thermotolerance
Source: Int J Mol Sci. 2024 Dec 31;26(1):287. doi: 10.3390/ijms26010287 (PMC11719701; doi:10.3390/ijms26010287)
Supplement: Supplementary file 1 [file ijms-26-00287-s001.zip › Supplementary figures.pdf]

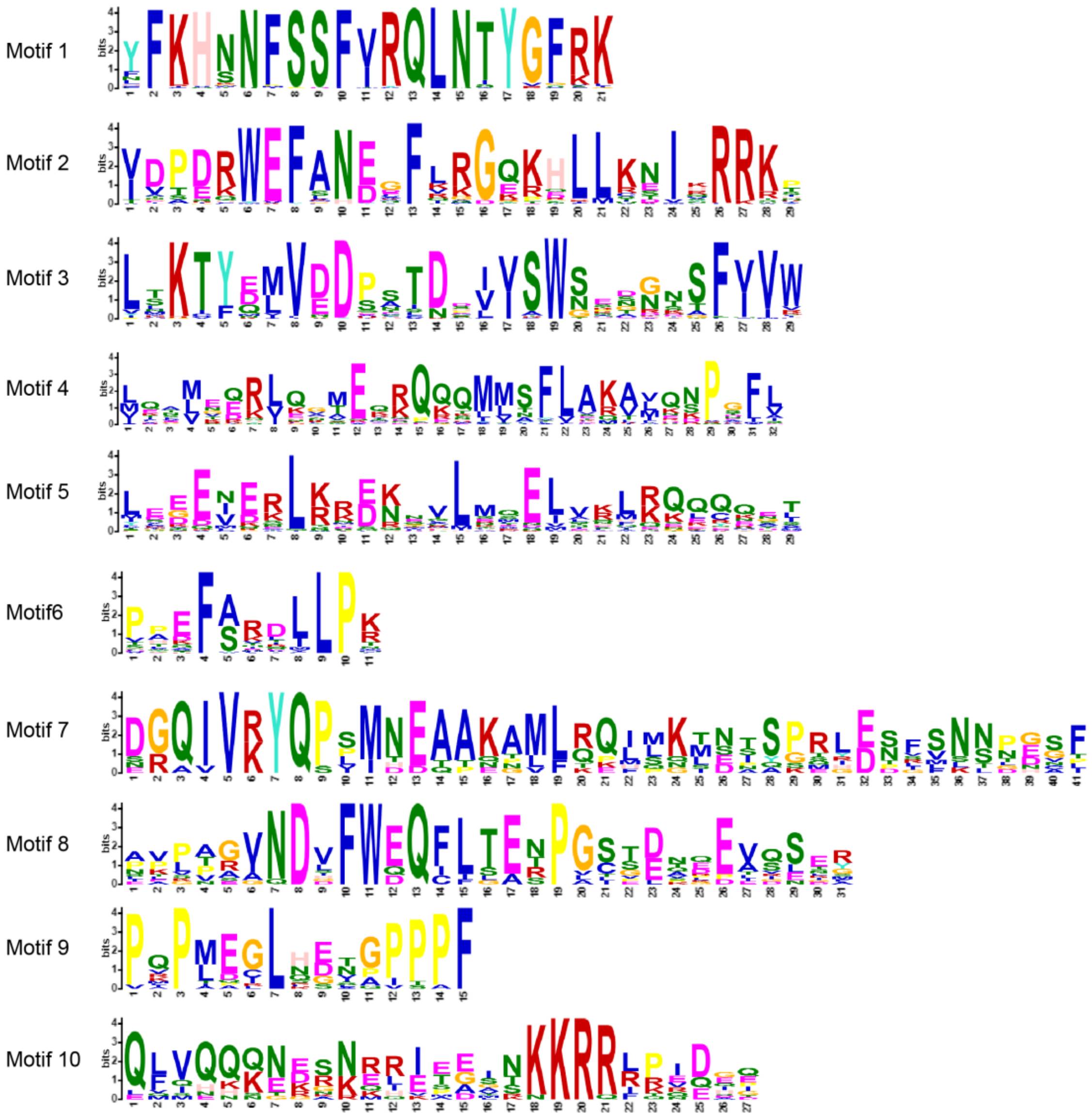

Supplemental Figure S1. The distribution and visualization of 10 motif identified in RcHsf proteins sequences.

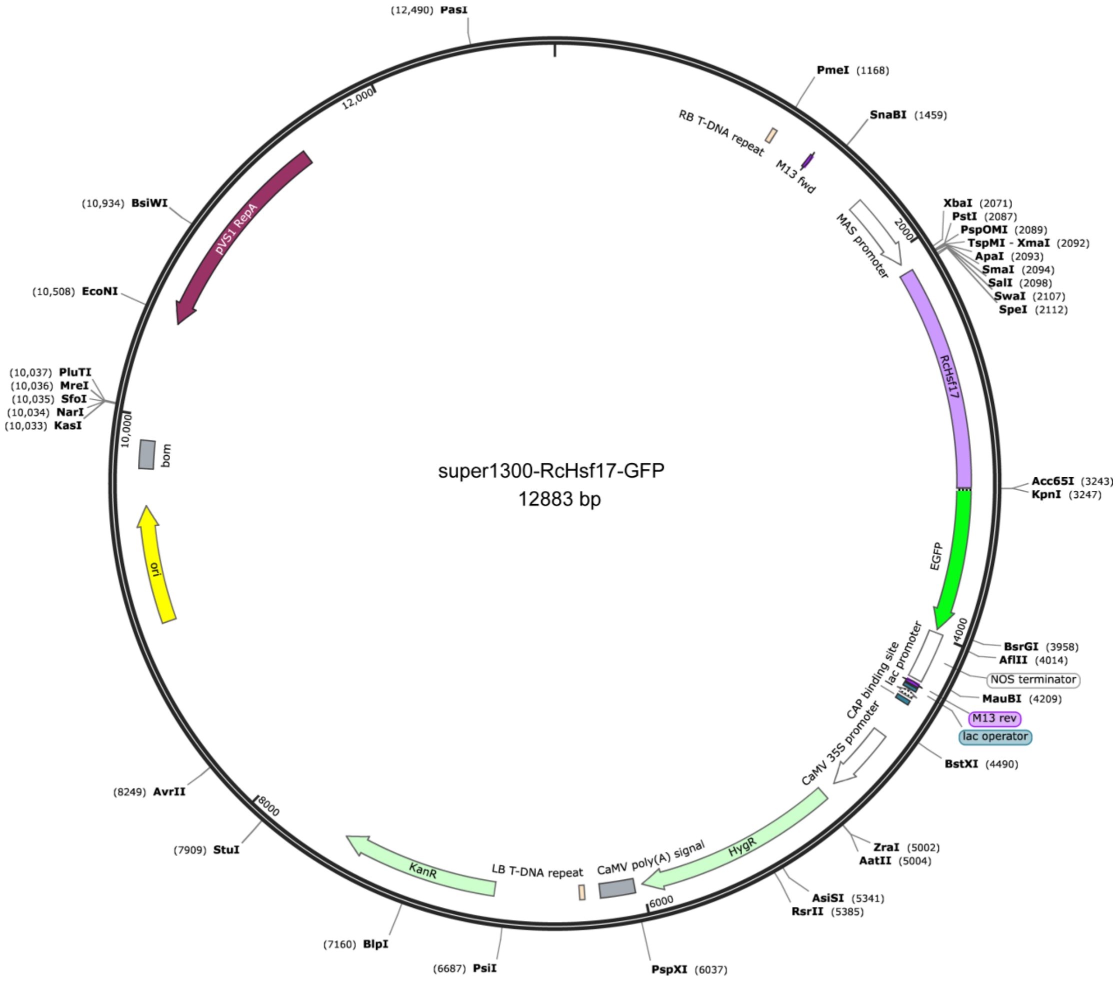

Supplemental Figure S2. The vector map of recombinant plasmid super1300-RcHsf17-GFP.

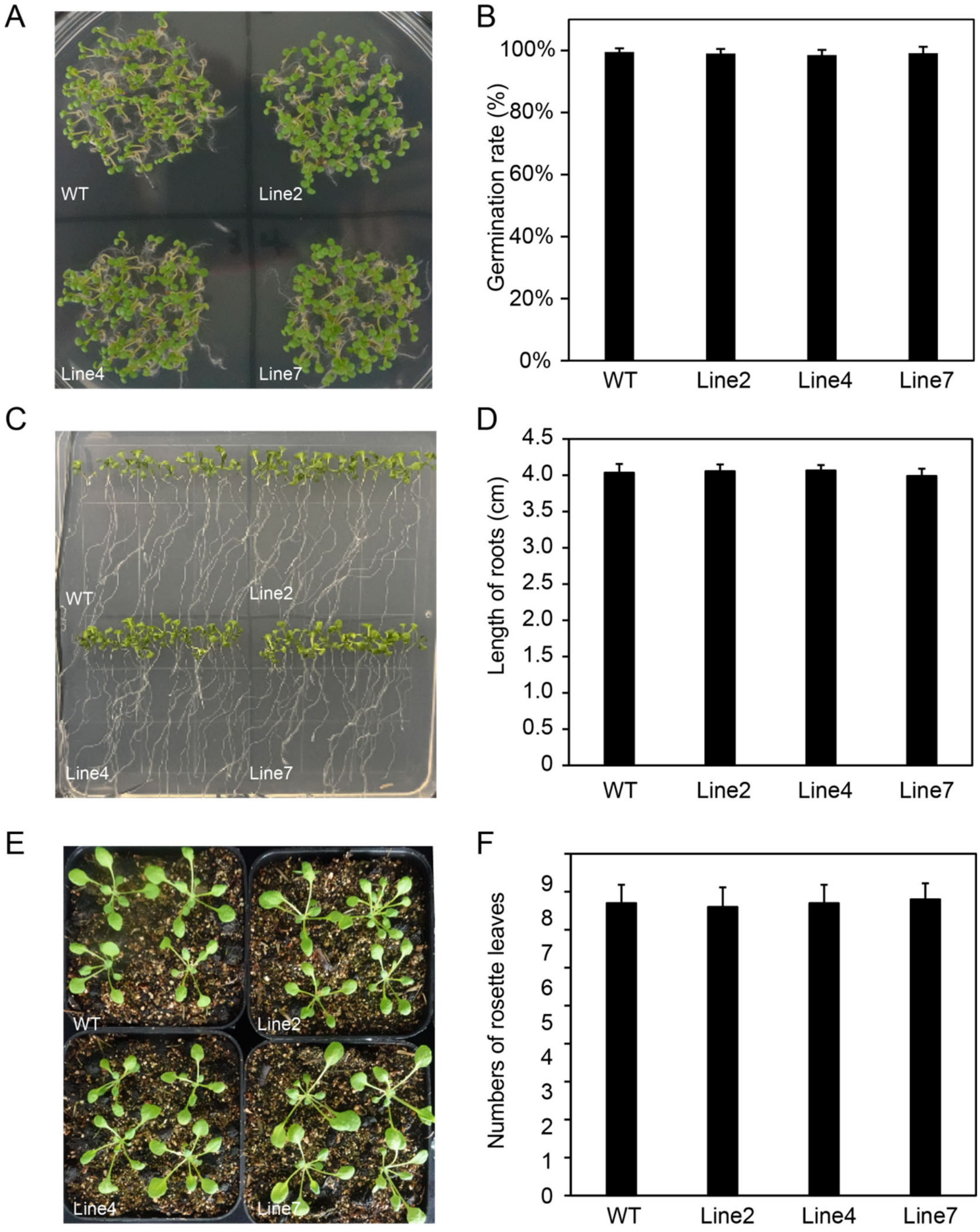

Supplemental Figure S3. The phenotype analyses of *RcHsf17* overexpression *Arabidopsis* seedlings; A: The phenotype of wild type and *RcHsf17* overexpression lines grown on 1/2 MS medium for 10 days. B, Germination rate of wild type and *RcHsf17* overexpression lines; C, The roots phenotype of roots of wild type and *RcHsf17* overexpression lines grown on 1/2 MS medium for 15 days; D, Statistics of roots length of wild type and *RcHsf17* overexpression lines. E, The phenotype of wild type and *RcHsf17* overexpression lines grown on soil for 15 days; F, Numbers of rosette leaves of wild type and *RcHsf17* overexpression seedlings. Data are means ±SD of three biological replicates.

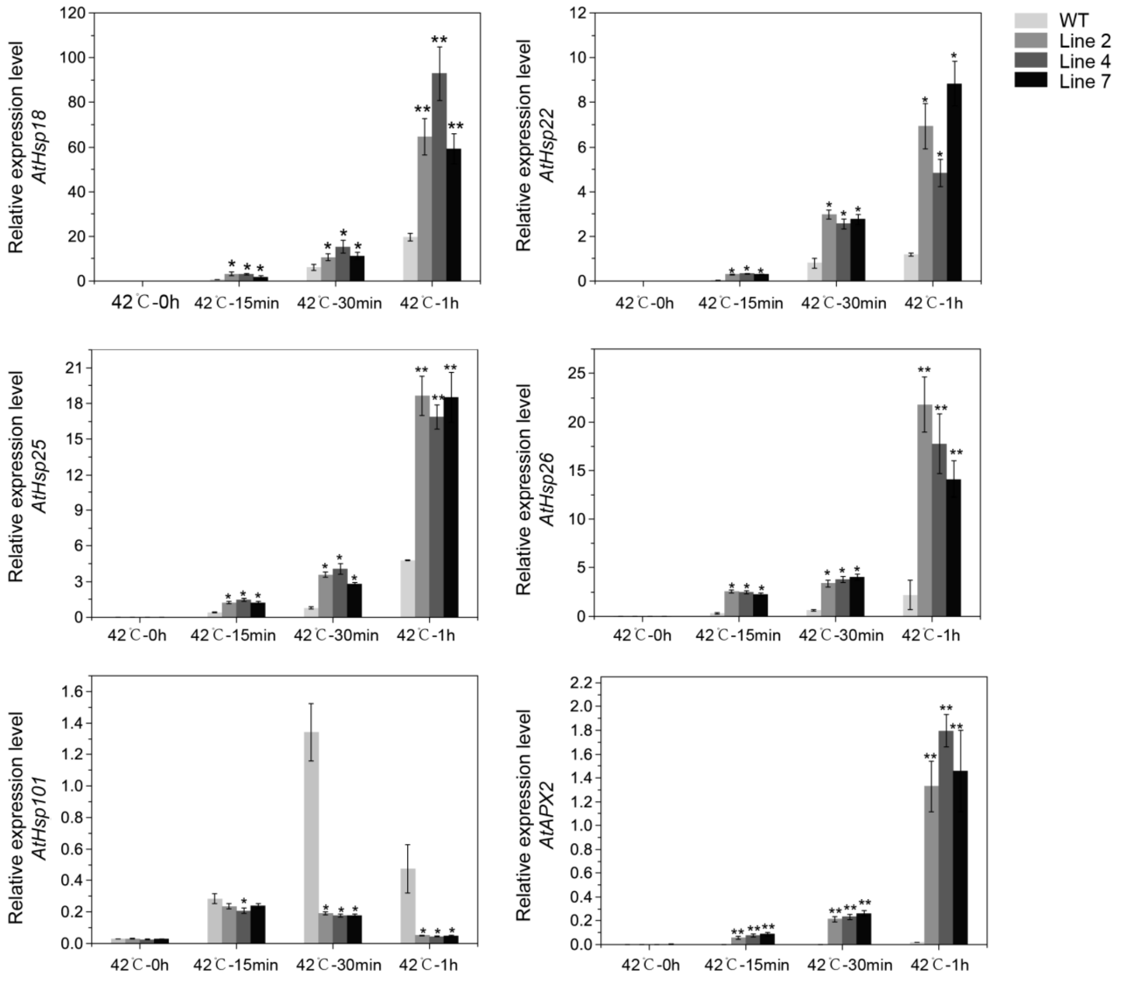

Supplemental Figure S4. Expression of related to high temperature response in *Arabidopsis*. The gene expression levels of 14-days-old wild type and transgenic *Arabidopsis* seedlings treated under 42°C for 15 min, 30 min and 1 h were detected by RT-PCR. Data were means ±SD of three biological replicates. The asterisks on the top of bars indicated significant differences between wild type and *RcHsf17* transgenic plants. * $p < 0.05$ by t-test,** $p < 0.01$ by t-test.
